# Cumulative reproductive costs on current reproduction in a wild polytocous mammal

**DOI:** 10.1002/ece3.4597

**Published:** 2018-11-14

**Authors:** Svenja B. Kroeger, Daniel T. Blumstein, Kenneth B. Armitage, Jane M. Reid, Julien G. A. Martin

**Affiliations:** ^1^ Institute of Biological & Environmental Sciences, School of Biological Sciences University of Aberdeen Aberdeen UK; ^2^ Department of Ecology and Evolutionary Biology University of California Los Angeles California; ^3^ The Rocky Mountain Biological Laboratory Crested Butte Colorado; ^4^ Ecology & Evolutionary Biology Department The University of Kansas Lawrence Kansas

**Keywords:** disposable soma theory, individual quality, life‐history strategies, long‐lived rodent, reproductive trade‐offs, resource allocation

## Abstract

The cumulative cost of reproduction hypothesis predicts that reproductive costs accumulate over an individual's reproductive life span. While short‐term costs have been extensively explored, the prevalence of cumulative long‐term costs and the circumstances under which such costs occur alongside or instead of short‐term costs, are far from clear. Indeed, few studies have simultaneously tested for both short‐term and cumulative long‐term reproductive costs in natural populations. Even in mammals, comparatively little is known about cumulative effects of previous reproduction, especially in species with high variation in offspring numbers, where costs could vary among successful reproductive events. Here, we quantify effects of previous short‐term and cumulative long‐term reproduction on current reproduction probability and litter size in wild female yellow‐bellied marmots (*Marmota flaviventer*) and test how these effects vary with age and between two contrasting environments. We provide evidence for cumulative long‐term effects: females that had both reproduced frequently and weaned large litters on average in previous years had decreased current reproduction probability. We found no evidence for short‐term reproductive costs between reproductive bouts. However, females weaned larger litters when they had weaned larger litters on average in previous years and had lower current reproduction probability when their previous reproductive success was low. Together these results suggest that, alongside persistent among‐individual variation, long‐term reproductive history affects current reproductive success.

## 1 INTRODUCTION

A central assumption of life‐history theory is that resources are limited, and thus trade‐offs are expected, where allocation to current reproduction reduces future reproduction and survival (Cody, 1966; Williams, 1966). However, such “costs of reproduction” can be difficult to quantify. A review of empirical studies of free‐living mammals reported mixed evidence of reproductive costs across traits and species and suggested that the likelihood of detecting costs in a given fitness component depends on a species’ life‐speed (Hamel et al., 2010). Specifically, short‐term reproductive costs on reproduction were more likely to be found in ungulates, which have high variance in reproductive parameters and low variance in survival (live slow), whereas reproductive costs on survival were more likely to be found in rodents (Hamel et al., 2010), which tend to exhibit low reproductive variability and high variance in survival (live fast; Gaillard & Yoccoz, 2003; also see Bleu, Gamelon, & Sæther, 2016 in birds). However, costs may not only occur in the short‐term, but could potentially only be detected after a certain amount of physiological damage has accumulated. Thus, due to competing demands of reproductive activities and somatic maintenance (Kirkwood & Rose, 1991), frequent reproduction could generate cumulative costs over an individual's reproductive life span (Moyes et al., 2006). In addition, reproductive costs could vary among successful reproductive events in species that can produce multiple offspring per reproductive event (i.e., polytocous species) and may only be expressed at old ages due to senescence (Descamps, Boutin, McAdam, Berteaux, & Gaillard, 2009) or under harsh environmental conditions (Tavecchia et al., 2005). The prevalence and magnitude of cumulative reproductive costs in natural age‐structured populations and their importance in shaping environment‐specific reproductive strategies alongside short‐term reproductive costs remain little understood.

When there are short‐term costs of reproduction, a reduction in current reproductive success is expected following a successful reproductive event in the previous year (Stearns, 1992). When there are cumulative long‐term costs of reproduction, future performance is expected to decrease following high previous reproductive allocation over several reproductive events. This expectation is also generally in line with the disposable soma theory of senescence, which posits that senescence rates depend on age‐related (early life vs. late life) resource allocation trade‐offs between reproduction and self‐maintenance (Kirkwood & Rose, 1991; Kirkwood, 1977).

In birds, long‐term reproductive costs on both future reproduction and survival appear to be common (western gulls, *Larus occidentalis*, Pyle, Nur, Sydeman, & Emslie, 2018; willow tits, *Parus montanus,* Orell & Belda, 2002; red‐billed chough, *Pyrrhocorax pyrrhocorax*, Reid, Bignal, Bignal, McCracken, & Monaghan, 2003; common guillemots, *Uria aalge*, Reed et al., 2008; great tits, *Parus major*, Bouwhuis, Charmantier, Verhulst, & Sheldon, 2010). For example, female common guillemots that had more chicks in early life had lower breeding success later in life (Reed et al., 2008). In mammals on the other hand, long‐term reproductive costs are less well supported. Long‐term costs were reported in bison (*Bison bison*, Green & Rothstein, 1991), northern elephant seals (*Mirounga angustirostris*, Sydeman, Huber, Emslie, Ribic, & Nur, 1991) and red deer (*Cervus elaphus*, Nussey, Kruuk, Donald, Fowlie, & Clutton‐Brock, 2006; Nussey, Kruuk, Morris, & Clutton‐Brock, 2007; Lemaître, Gaillard, Pemberton, Clutton‐Brock, & Nussey, 2014 but see Moyes et al., 2006). However, studies on different ungulate species (bighorn sheep, *Ovis canadensis*, Bérubé, Festa‐Bianchet, & Jorgenson, 1999; fallow deer, *Dama dama*, McElligott & Haydon 2000; red deer, *C. elaphus*, Moyes et al., 2006; mountain goats, *Oreamnos americanus*, Panagakis, Hamel, & Côté, 2017), gorillas (*Gorilla gorilla*, Robbins, Robbins, Gerald‐Steklis, & Steklis, 2006), killer whales (*Orcinus orca*, Ward, Parsons, Holmes, Balcomb, & Ford, 2009), Antarctic fur seals (*Arctocephalus gazella*, Arnould & Duck, 1997) and red squirrels (*Tamiasciurus hudsonicus*, McAdam, Boutin, Sykes, & Humphries, 2007) found no evidence for long‐term costs. Although there are too few studies to draw any conclusions, it appears that in long‐lived mammals, not only short‐term but also long‐term reproductive costs are more commonly observed on future reproduction than on future survival (Hamel et al., 2010; Lemaître et al., 2015). Again, the reverse would be expected for short‐lived mammals; however, even fewer studies on cumulative reproductive costs exist in such systems. Overall, long‐term reproductive costs are less frequently detected in mammals than in birds, and it is not yet understood how common cumulative costs are in mammals, or under which circumstances they are most likely to be observed.

One challenge with testing for cumulative long‐term reproductive costs is to quantify previous reproductive allocation. Previous empirical studies have used a variety of reproductive measures. For example, parturition success in “early life” (Panagakis et al., 2017), or in all previous years (Sydeman et al., 1991), the number of copulations in the first two years of social maturity (McElligott & Hayden, 2000) and the number of previous breeding attempts up to a certain age (successful or not; Pyle et al., 2018; Orell & Belda, 2002). Other measures of reproductive traits relate to the number of offspring produced, for example, the total number of young produced (e.g., Bouwhuis et al., 2010; Bérubé et al., 1999), clutch size and fledgling success at certain ages (Reid et al., 2003), and the total number of offspring produced, divided by years since sexual maturity (Moyes et al., 2006). Notably, in birds, a vast proportion of studies that have tested for long‐term reproductive costs are on species that can lay multiple eggs per clutch (but see Reed et al., 2008; Aubry, Koons, Monnat, & Cam, 2009). In mammals on the other hand, studies that tested for long‐term costs were mainly on monotocous species, which give birth to a single offspring per reproductive event or very rarely two (e.g., northern elephant seals, *M. angustirostris*, Sydeman et al., 1991; red deer, *C. elaphus*, Moyes et al., 2006; Nussey et al., 2006; Nussey et al., 2007; killer whales, *O. orca*, Ward et al., 2009). However, cumulative costs may also depend on the number of offspring weaned, not just the number of reproductive events.

Indeed, lactation in female mammals is energetically highly expensive (Clutton‐Brock, Albon, & Guinness, 1989; Gittleman & Thompson, 1988; Oftedal, 1985), and based on this, one might expect greater litter sizes to increase reproductive costs associated with each reproductive event. Thus, some knowledge gaps that require attention in relation to reproductive costs are as follows: first, to gain a better understanding of the prevalence and relative importance of short‐term and long‐term costs in mammals, we need further studies on different species that test for long‐term effects in addition to short‐term effects of previous reproduction on future performance. Second, using polytocous species and defining measures for both previous reproductive frequency and number of offspring would be particularly interesting to elucidate which part of reproduction is actually costly in these species: reproduction per se, the number of offspring produced, or an interaction between the two?

However, a number of factors could make it difficult to detect reproductive costs. Reproductive allocation and/or costs of reproduction could be age‐related, such that expression of costs depends on an individual's age class (e.g., primarily in young and old individuals; Descamps et al., 2009). Environmental heterogeneity could also mask reproductive costs, and it may be difficult to detect costs in resource‐rich environments (Ricklefs & Cadena, 2007), and/or costs could be higher in unfavorable conditions (Reed et al., 2008). Finally, reproductive costs could be masked by among‐individual variation (Wilson & Nussey, 2010), because individuals vary in resource acquisition (van Noordwijk & de Jong, 1986). While randomized experiments can be used to minimize bias resulting from individual and environmental heterogeneity, an experimental approach may not be feasible depending on the system of interest (Hamel et al., 2010). Approaches using longitudinal data at the individual level allow to control for and estimate among‐individual variation across age classes and environments (Cam, Link, Cooch, Monnat, & Danchin, 2002; Hamel et al., 2010).

In this study, we use long‐term data on yellow‐bellied marmots (*Marmota flaviventer*), a polytocous, hibernating rodent (Frase & Hoffmann, 1980), to quantify both short‐term and cumulative long‐term effects of female previous reproduction on current reproduction, and we test how these effects vary according to age and environmental conditions. Yellow‐bellied marmots are a moderately long‐lived species, with females reaching life spans of up to 14 years in the wild (Kroeger, Blumstein, Armitage, Reid, & Martin, 2018). While they are shorter‐lived than many ungulate species, yellow‐bellied marmots are longer‐lived than most other rodent species in previous studies of reproductive costs (Hamel et al., 2010), and we considered them to be a “long‐lived” species.

Most previous empirical studies that estimated long‐term reproductive costs were carried out in the context of early‐late life trade‐offs and the disposable soma theory of senescence, thus related measures of previous reproductive performance were restricted to a defined time period (“early life,” e.g., up to age 5, willow tits, *P. montanus*, Orell & Belda, 2002; between 3 and 6 years, mountain goats, *O. americanus*, Panagakis et al., 2017). Consequently, these studies were testing for a decrease in performance after a defined point in “late life” (i.e., after the defined “early life” period). Here, we quantified long‐term costs continuously, over the entire reproductive life span, without a restricted view on what constitutes early or late life. As marmots in our study population have been systematically monitored from birth to death throughout every active season since 1962 (Armitage, 2014), this study system has detailed longitudinal data on reproductive events and litter sizes for females at all ages. In addition, study individuals live in one of two contrasting environments that differ in elevation and hence phenology and ecology (Blumstein, Im, Nicodemus, & Zugmeyer, 2004; Kilgore & Armitage, 1978), which allows testing whether relationships between previous and current reproduction differ between environments.

As we considered yellow‐bellied marmots to be a long‐lived species, we hypothesized that we should observe reproductive costs on current reproduction (Hamel et al., 2010; Stearns, 1992). Contrary to most previous studies, we tested for short‐term and long‐term reproductive costs simultaneously. We used previous reproductive frequency and average litter size as measures of previous cumulative reproductive allocation and tested the hypotheses that females incurred both short‐term and cumulative long‐term costs, following either one year of successful weaning or following greater cumulative reproductive allocation in all the previous years, respectively. We specifically hypothesized that the combination of reproducing frequently and having large numbers of offspring incurs long‐term costs.

As physiological function tends to decrease with increasing age (i.e., senescence; Ricklefs, 2008), we also hypothesized that reproductive costs are higher in older individuals, especially in females with greater previous reproductive allocation. Finally, we tested for effects of environmental conditions on reproductive costs. Reproductive strategies can vary along elevational gradients, with higher elevation environments commonly presenting harsher conditions than lower elevation environments (e.g., Bears, Martin, & White, 2009). As reproductive costs may be more likely detected under unfavorable conditions (e.g., Tavecchia et al., 2005), we hypothesized that reproductive costs are more likely to be observed in the higher elevation environment than the lower elevation environment.

## 2 METHODS

### 2.1 Study area and marmot life‐history

We studied a yellow‐bellied marmot population around the Rocky Mountain Biological Laboratory (RMBL; approximately 2,900 m elevation), over a 5 km stretch of the Upper East River Valley, Colorado. The study area includes two distinct regions: “up‐valley” and “down‐valley”. Marmot movement between the two regions is rare, and individuals included in our analyses lived either up‐valley or down‐valley throughout their entire lives. The “up‐valley” region is at approximately 165 m higher elevation than “down‐valley” and is characterized by later snowmelt, and hence later onset of vegetation growth and marmot emergence from hibernation (Blumstein, 2009; Blumstein et al., 2004; Monclús, Pang, & Blumstein, 2014). As the first killing frosts occur at similar times in both valley regions, the overall vegetation growing season is shorter up‐valley (van Vuren & Armitage, 1991). Females in both regions are sexually mature at two years of age. Mating occurs in May, following emergence from hibernation, and between mid‐May and mid‐June, successfully reproducing females give birth underground to a single litter of 1–10 pups (Blumstein, 2009; Frase & Hoffmann, 1980). Offspring are nursed for 25–35 days and are weaned and fully independent upon emerging (Armitage, 2014; Nee, 1969).

### 2.2 Female reproduction data collection

Between 1962 and 2014, we trapped adult females fortnightly from mid‐May to mid‐September. Individuals were identified via uniquely numbered ear tags, given at first capture. Because litters are born in underground burrows, the earliest access to pups was at first emergence in June or July. Weekly observations of all colonies allowed detection of pup emergence and estimation of litter sizes. Pups were captured, tagged and dorsally marked with nontoxic fur dye within 1–2 weeks of emerging. Fur marks made individuals identifiable from afar, thus newly emerged pups were distinguishable from previously caught ones during colony observations. Very few, if any, emerged pups were missed since all colonies within the study area were very closely monitored with near‐daily observations during the pup emergence season. In adults, multistate mark‐recapture analyses estimated the annual recapture probability to exceed 98% (Ozgul, Armitage, Blumstein, & Oli, 2006; Ozgul, Oli, Olson, Blumstein, & Armitage, 2007).

Maternity was assigned based on behavioral observations. From 2000 onwards, assigned maternities were additionally confirmed via genetic analyses using 8–12 microsatellite loci at 95% trio confidence level (further details in Blumstein, Lea, Olson, & Martin, 2010) and were congruent in 98% of cases. A female was classified as having reproduced in a given year if she had at least one weaned offspring assigned to her; otherwise she was classified as nonreproducing. Since lactation is the most energetically expensive component of reproduction in female mammals (Clutton‐Brock et al., 1989; Oftedal, 1985), the number of weaned offspring likely captures the vast majority of reproductive costs per born litter, even if some unobserved pups died early underground.

### 2.3 Analyses

#### 2.3.1 Quantifying costs of previous reproduction

We defined and used three metrics of previous reproduction: one quantifying short‐term reproduction and two quantifying cumulative long‐term reproduction. To quantify short‐term reproduction, females were initially classified as having reproduced last year or not (RLY, a two‐level factor). Due to collinearity issues between the number of reproductive events and number of weaned offspring in previous years (*r* = 0.90, Figure S1), and of these variables with female age (*r*_(Nr.Reprod.)_ = 0.57; *r*_(Nr.pups)_ = 0.48; Figure S1), to quantify cumulative long‐term reproduction, we decided not to use the number of previous reproductive events and of weaned offspring as explanatory variables per se. Instead, we first used a similar measure to Nussey et al. (2007). We calculated previous reproductive frequency (PRF), defined as the proportion of years in which a female weaned pups since her first successful reproduction. This variable was calculated for each female in each year, as the total number of previous years in which a female successfully weaned pups, divided by the number of years since her first weaned litter (excluding the current year). Second, we calculated a measure related to previous number of offspring, defined as the mean number of pups weaned across all previous successful reproductive events (previous average litter size, PALS). This variable was calculated for each female in each year, as the total number of previously weaned pups, divided by the total number of previous successful reproductive events.

The variables in both ratios (PRF: previous number of reproductive events/previous years of reproductive activity; PALS: previous number of weaned offspring/previous number of successful reproductive events), are isometrically related (linear relation with intercept at 0; Packard & Boardman, 1988). Thus, the ratio transformations should successfully standardize the numerator for the effects of the denominator, and there should be no spurious correlations associated with the use of ratios in our models (Kronmal, 1993; Packard & Boardman, 1988).

#### 2.3.2 Statistical models

To quantify relationships between previous reproduction and current reproduction, we fitted two generalized linear mixed‐effects models (GLMMs). The first estimated a female's probability to reproduce in the current year, using a binomial distribution with logit link. The second estimated the weaned litter size in the current year given that a female reproduced, using a Poisson distribution with log link.

Fixed effects in both models included the short‐term previous reproduction variable RLY, the two long‐term previous reproduction variables PRF and PALS, second‐degree polynomial age effects (hereafter: “linear and quadratic age”), age at first reproduction (AFR), valley (a two‐level factor: up vs. down), the number of mature daughters living in the same colony as the mother, and interactions between those variables.

Specifically, to test the hypotheses that females incurred short‐term and/or cumulative long‐term costs of previous reproduction on current reproduction, we fitted the short‐term (RLY) and long‐term (PRF and PALS) previous reproduction variables in both models. To test whether costs are only expressed when individuals have both reproduced frequently and weaned large litters on average, we fitted a two‐way interaction between the two long‐term previous reproduction variables (PRF and PALS).

To test the hypothesis that reproductive costs increase in older individuals, we fitted two‐way interactions between linear and quadratic age and all three previous reproduction variables (RLY, PRF and PALS). To test the hypothesis that costs differ between environments, we fitted two‐way interactions between valley and all three previous reproduction variables.

Since age at first reproduction (AFR) affects the number of potentially available reproductive seasons over which costs may accumulate, we also fitted age at first weaned litter to account for differences in onset of reproduction. Further, large matriline sizes have previously been found to negatively affect female yellow‐bellied marmot reproductive success (Armitage & Schwartz, 2000). Thus, to control for potential effects of mother‐daughter competition on female current reproduction probability and litter size, we included a fixed effect of the number of sexually mature daughters living in the same colony as the mother in each year (“Mat_daughters”). Pearson correlations between the number of mature daughters and previous cumulative reproduction variables were low (r_(PRF)_ = 0.20; r_(PALS)_ = 0.09), because females only reach sexual maturity at 2 years or older.

Analyses included only females of known age (i.e., first captured as a pups) and with completely known reproductive histories from age at sexual maturity to death. To avoid selective disappearance biases in the data structure, all individuals from nonextinct cohorts were excluded, with the exception of two nearly extinct cohorts (2 of 18, and 1 of 24 individuals still alive). To allow for simultaneous testing of short‐term and cumulative long‐term effects of previous reproduction on current reproduction, analyses were further restricted to females with at least three years of reproductive activity (years since first successfully weaned litter, including the current year). Every female therefore had at least two years of previous reproductive activity.

To account for nonindependence of repeated measures, random individual identity, year, and cohort effects were also fitted. We found little evidence for overdispersion of current reproduction probability (residual deviance/residual degrees of freedom ratio = 1.1), and little evidence of underdispersion of current litter size (ratio = 0.8), thus it was not necessary to take them into account.

Results are presented for full models, including significant and nonsignificant effects (Whittingham, Stephens, Bradbury, & Freckleton, 2006), except nonsignificant interactions (*p* > 0.05) were backwards eliminated as they may bias other estimates (Engqvist, 2005). Age effects were fitted as orthogonal polynomials up to the second order (this removes the correlation between linear and quadratic age so that each can be interpreted independently; Crawley, 2007). To facilitate interpretability of coefficients and allow comparison of the relative influence of each explanatory variable, continuous predictors were centered on 0 by subtracting the variable mean from each observed value and scaled by dividing them by 2 standard deviations (Gelman, 2008). Models were fitted in R 3.5.1. (R Core Team, 2018) using the glmer function in the lme4 package (Bates, Mächler, Bolker, & Walker, 2015).

Results remained quantitatively similar when we refitted all models including last year litter size (LYL) instead of RLY (Tables S1 and S2). Fitting lagged values of a trait (e.g., trait at *t*−1) as fixed effects in mixed models of the same trait (i.e., trait at *t*) can lead to biases in model parameters by violating model assumptions of independence. That is because the random intercept is not statistically independent of the lagged dependent variable as it directly affects it (Rabe‐Hesketh & Skrondal, 2012). Thus, estimating short‐term costs of reproduction could be problematic and provide biased results, for example, when estimating the probability to reproduce this year as a function of previous year reproduction (RLY). However, models fitted excluding RLY yielded quantitatively similar results as models including it (Tables S3 and S4).

Another factor to consider is that maternal body mass in spring affects current reproduction, but conversely reproduction also affects body mass: lactating females allocate resources to their pups and start accumulating fat reserves later than nonreproducing females (Armitage, 2014, pp. 98–100). Since we were interested in the trade‐off between previous and current reproduction and how this varies with age and between environments rather than the mechanism by which the trade‐off occurs, body mass was not included in our current models.

## 3 RESULTS

### 3.1 Data structure

Lifelong reproductive histories of females that had been reproductively active for at least three years were known for 108 individuals, born in 34 different years between 1962 and 2007 (Figure S2a). Data on current reproduction were available for every year between 1966 and 2014 (Figure S2b). Data collected past 2014 were not included as the majority of individuals from these recent cohorts were still alive. Observations included females aged 4 to 14 years (mean = 6.8, median = 6.0, IQR = 5.0–8.0), living either up‐valley (62 individuals, 187 observations) or down‐valley (46 individuals, 152 observations). Out of all 339 observed reproductive events, 183 were successful with litter sizes of 1 to 9 pups (mean = 4.3, median = 4.0, IQR = 3.0–6.0; Figure S3a). In 184 of 339 cases, females had reproduced successfully in the previous year (93 down‐valley, 91 up‐valley). Full details of sample sizes of observations of reproduction and litter sizes at each age and in both valley regions are shown in Figure S4.

The proportion of years in which a female weaned pups (PRF) ranged from 0.1 to 1.0 (mean = 0.7, median = 0.7, IQR = 0.5–1.0; Figure S3b), and previous average litter sizes (PALS) ranged from 1 to 8 pups (mean = 4.0, median = 4.0, IQR = 3.0–5.0; Figure S3c). Structural relationships between PRF and years of reproductive activity and PALS and the number of successful reproductive events are shown in Figure S5. Collinearities among all three previous reproduction variables were explored and showed that PRF and PALS explained 5% of the variation in each other (*R* ^2^ = 0.05; Pearson correlation coefficients are shown in Figure S6). In addition, variance inflation factors for predictors in our models were all below 3, and thus below the commonly used threshold value of 4 (O'Brien, 2007), indicating only low to moderate multicollinearity and justifying the inclusion of PRF and PALS within the same model. Maternal age at first weaning ranged from 2 to 6 years (mean = 3.0, median = 3.0, IQR = 2.0–3.0) and did not affect current reproduction probability or litter size of females in our dataset (over 90% of females had weaned their first litter by age 4).

### 3.2 Effects of previous reproduction on current reproduction

There were no short‐term effects of reproduction the previous year (RLY) on either current reproduction probability (Table 1) or current litter size (Table 2). In both models, interactions of RLY with age and valley were not significant (Tables S5 and S6).

**Table 1:** Generalized linear mixed‐effects model estimating effects of previous short‐term reproduction (RLY, reproduced last year) and cumulative long‐term reproduction (PRF, previous reproductive frequency; and PALS, previous average litter size), age, valley, age at first reproduction (AFR), and number of sexually mature daughters living in the same colony (Mat_daughters) on current reproduction probability of female yellow‐bellied marmots

Fixed effect	Estimate	*SE*	*z*	*p*‐value
Intercept	0.22	0.35	1.04	0.296
RLY[yes]	0.00	0.42	0.01	0.995
PRF	0.55	0.92	1.12	0.263
PALS	0.41	0.11	1.40	0.162
**PALS × PRF**	**−1.58**	**0.41**	**−2.60**	**0.009**
Age	−0.44	3.18	−1.26	0.207
Age^2^	−0.38	2.66	−1.30	0.192
Valley[up]	−0.27	0.31	−0.87	0.383
AFR	0.16	0.17	0.49	0.625
Mat_daughters	0.42	0.13	1.35	0.177

Estimated effects sizes are reported with standard errors (*SE*) and *z*‐test statistics (*z*). Significant terms are shown in bold. Eliminated interaction terms are shown in Table S5. The reference levels for valley and RLY are [down] and [no], respectively. Random effects variances are 0.00, 0.69, and 0.11 for “female identity,” “year observed,” and “cohort,” respectively.

**Table 2:** Generalized linear mixed‐effects model estimating effects of previous short‐term reproduction (RLY, reproduced last year) and cumulative long‐term reproduction (PRF, previous reproductive frequency; and PALS, previous average litter size), age, valley, age at first reproduction (AFR), and number of sexually mature daughters living in the same colony (Mat_daughters), on current litter size of female yellow‐bellied marmots

Fixed effect	Estimate	*SE*	*z*	*p*‐value
**Intercept**	**1.45**	**0.09**	**16.20**	**<0.001**
RLY[yes]	−0.03	0.12	−0.30	0.767
PRF	0.15	0.26	1.17	0.242
**PALS**	**0.18**	**0.03**	**2.15**	**0.032**
Age	−0.03	0.69	−0.29	0.770
Age^2^	−0.04	0.55	−0.47	0.638
Valley[up]	−0.09	0.09	−1.00	0.320
AFR	−0.16	0.05	−1.64	0.101
Mat_daughters	0.02	0.04	0.20	0.839

Estimated effects sizes are reported with standard errors (*SE*) and *z*‐test statistics (*z*). Significant terms are shown in bold. Eliminated interaction terms are shown in Table S6. The reference levels for valley and RLY are [down] and [no], respectively. Random effects variances are 0.00, 0.00, and 0.01 for “female identity,” “year observed,” and “cohort,” respectively.

**Figure 1:**
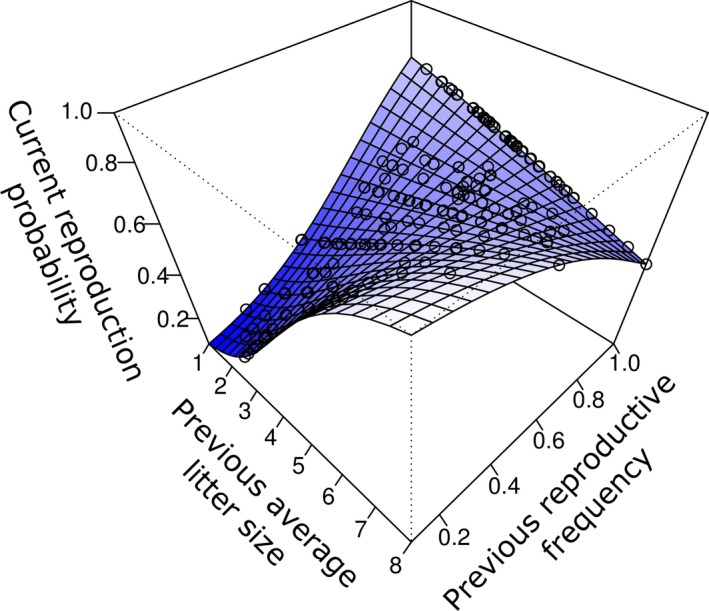
Current reproduction probability in female yellow‐bellied marmots in relation to two measures of cumulative long‐term reproduction: previous average litter size and previous reproductive frequency. The surface shows model predictions; darker shading indicates lower values of current reproduction probability. Points show distribution of the data on the predicted surface

There were cumulative, long‐term effects of previous reproduction on current reproduction. Specifically, there was a significant interactive effect of the two cumulative reproductive variables (PRF and PALS) on current reproduction probability (Table 1). Females which both reproduced frequently (high PRF), and had high average litter sizes in previous years (high PALS), had lower reproduction probabilities in the current year (Figure 1; Figure S7). Females that both reproduced at low frequency (low PRF), and had low average litter sizes in previous years (low PALS), also had lower reproduction probabilities in the current year (Figure 1; Figure S7). Females with low PALS and high PRF and with high PALS and low PRF had high current reproduction probabilities (Figure 1; Figure S7). Interactions of PRF and PALS with age and valley were not significant (Table S5).

**Figure 2:**
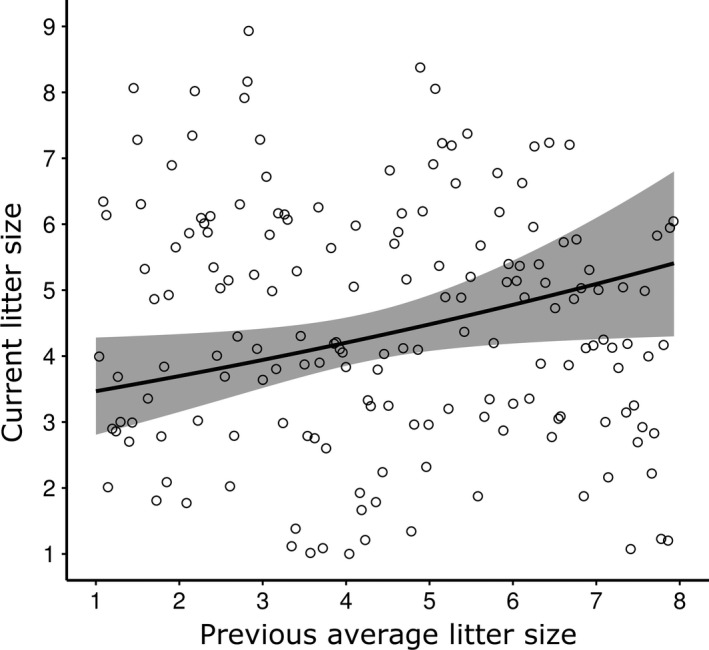
Current litter size of female yellow‐bellied marmots in relation to their previous average litter size. The line shows model predictions, and points show the distribution of the raw data

There was also a significant main effect of PALS on current litter size (Table 2; Figure 2), showing that reproducing females with high average litter sizes in previous years weaned larger litters in the current year. Interactions of PRF and PALS with age and valley and of PRF with PALS were not significant in relation to current litter size (Table S6).

There were no effects of age or valley on current reproduction probability or litter size (Tables 1 and 2). Further, there was no effect of the number of sexually mature daughters living in the same colony as the mother; thus, the decrease in reproduction probability in females with both high PRF and PALS could be interpreted as reproductive cost rather than competition of highly reproducing females with their daughters.

## 4 DISCUSSION

The importance of cumulative long‐term reproductive costs alongside short‐term costs, and how such costs vary with age and under different environmental conditions, is little explored in wild polytocous mammals. Although we did not find any evidence for short‐term effects of reproduction from one year to the next, we present evidence of cumulative long‐term effects of previous reproduction on current reproduction in a natural population of yellow‐bellied marmots. Reproduction probability decreased in females with both high previous reproductive frequencies and high average litter sizes. However, individuals with higher average litter sizes in previous years also weaned larger litters in the current year. Our results thus support both cumulative reproductive costs and persistent among‐individual differences in reproductive success (also referred to as “quality differences”; Wilson & Nussey, 2010). We did not find any evidence that effects of cumulative reproductive allocation vary with age or among environments. Our study suggests that reproduction in long‐lived polycotous mammals depends at least in part on individuals’ previous reproductive history.

### 4.1 Short‐term costs

Reproduction in the previous year was predicted to reduce reproduction probability or litter size in the current year. However, we found no association between RLY and current reproductive success, and thus no evidence for short‐term reproductive costs on current reproduction. Reproductive costs on current reproduction are expected to be more likely to be detected in longer‐lived rather than shorter‐lived mammals (Hamel et al., 2010). However, the probability of detecting reproductive costs may additionally depend on the stage of the reproductive cycle when reproduction is compared between individuals (e.g., conception, parturition and weaning). While female lactation is typically considered the most expensive stage of the mammalian reproductive cycle (Clutton‐Brock et al., 1989; Gittleman & Thompson, 1988), short‐term reproductive costs were less frequently detected in relation to weaning success than in relation to parturition success across various short‐ and long‐lived mammal species (Hamel et al., 2010). One possible explanation is that reproductive costs are more difficult to detect at later stages of the reproductive cycle because of among‐individual variation in reproduction. Some females are more likely to consistently raise offspring to weaning age (Hamel, Côté, Gaillard, & Festa‐Bianchet, 2009), thus leading to lower variation in reproductive output at weaning than at earlier stages (Hamel et al., 2010). Studies in closely related species show mixed results for short‐term effects of previous reproduction. A study in female hoary marmots (*Marmota caligata*) similarly found no effects of successfully weaning a litter in the previous year on current reproduction probability and survival (Patil, Karels, & Hik, 2015). In contrast, short‐term reproductive costs on breeding probability were found in Alpine marmots (*Marmota marmota*) and Olympic marmots (*Marmota olympus*), in relation to previous weaning and previous parturition success, respectively (Barash, 1973; Hackländer & Arnold, 1999). It is surprising that in the yellow‐bellied marmot, a species with high energetic requirements and limited time to gain fat reserves, current reproductive success apparently did not differ between females that did and did not wean pups in the previous year. Besides among‐individual differences, a likely explanation is that individuals may be able to recover physiological costs of reproduction during the active season (Patil et al., 2015). In addition, females may incur indirect reproductive costs, reflected as decreased offspring performance (sensu Hamel et al., 2010).

### 4.2 Cumulative long‐term costs

Females with high average litter sizes in previous years also weaned larger litters in the current year. Positive associations between current and future reproduction are commonly reported in long‐lived animals (Hamel et al., 2010), raising the question whether reproductive costs are masked by among‐individual differences in fitness (Hamel et al., 2009; Weladji et al., 2008). However, we accounted for differences in reproductive success among individuals by fitting random female effects in our models, and in addition to the positive association between previous average litter size and current litter size, we also found negative associations between different measures of previous cumulative and current reproduction (discussed below). Positive associations between previous and current litter size are likely due to persistent individual differences in state, involving differences in resource acquisition and allocation (McNamara & Houston, 1996; van Noordwijk & de Jong, 1986), which determine reproductive success. This is supported by results from a previous study in yellow‐bellied marmots, which found a positive effect of previous year reproduction on current reproduction probability (Nuckolls, 2010), and by studies in other systems (e.g., Alpine chamois, *Rupicapra rupicapra*, Tettamanti, Grignolio, Filli, Apollonio, & Bize, 2015; wood ducks, *Aix sposa*, Kennamer, Hepp, & Alexander, 2016). Our results thus suggest that previous weaning experience indicates persistent among‐individual differences in resource acquisition and allocation.

Interestingly, we detected a decrease in current reproduction probability in females that had both weaned larger litters and reproduced frequently in previous years (i.e., high‐high), suggesting cumulative reproductive costs. If lactation was the major reproductive cost and was independent of litter size, only frequent reproduction may lead to detectable costs. A study in bison, a monotocous species, suggested that maternal allocation per se may be limited (i.e., whether or not a female allocates to reproduction), but not the amount allocated if a female does reproduce (Hamel, Craine, & Towne, 2012). However, in polytocous mammals, it is more likely that variation in litter size may be due to among‐individual differences in reproductive success (as suggested above), in which case again costs may only be detected when females reproduce frequently.

Females with both small litter sizes and low previous reproductive frequencies (i.e., low‐low) had decreased current reproduction probabilities. Again, this is consistent with the idea that previous weaning experience indicates persistent among‐individual differences in acquisition and utilization of resources, as a result of which some females consistently have low values for reproductive traits. Individuals with smaller litters and high reproductive frequencies (i.e., low‐high) could also be expected to be less successful. However, our results do not support this expectation: generally, individuals appear to have either intermediate or low values for both cumulative reproductive measures, and individuals that reproduce often but wean small litters (high‐low) or reproduce less often but wean larger litters (low‐high) have higher current reproduction probabilities than either of the other two groups (i.e., high‐high or low‐low). Mothers can reduce allocation to their young through decreased milk production (Fite et al., 2005), and thus costs could also be transferred from mother to offspring (Martin & Festa‐Bianchet 2010; Hodges, Bowers, Thompson, & Sakaluk, 2015). In female house mice (*Mus musculus*), for example, offspring in larger litters had lower weaning weights that offspring from smaller litters (König, Riester, & Markl, 1988). Field experiments investigating short‐term year‐to‐year reproductive costs of previous litter size commonly report that females do not trade off future performance against number of offspring (e.g., ground squirrels, Neuhaus, 2000; Skibiel, Speakman, & Hood, 2013; but see Koivula, Koskela, Mappes, & Oksanen, 2003 in bank voles). Mothers likely adjust the number and size of offspring contingent on their capability to wean young (e.g., Columbian ground squirrels, *Spermophilus columbianus*, Neuhaus, 2000; Alpine marmots, *M. marmota*, Berger, Lemaître, Gaillard, & Cohas, 2015), thus optimizing their lifetime reproductive success (optimal investment hypothesis, Morris, 1992). Our results suggest that the same individual optimization strategy is maintained throughout life, as the number of offspring does not directly reduce either short‐term or long‐term future reproduction. Furthermore, we did not find evidence that cumulative effects differ between the higher and lower elevation environment, suggesting that individuals follow the same optimization strategy at both elevations; alternatively, there was not enough power to detect differences between elevations.

A decrease in reproductive success with age in individuals with high previous reproductive frequencies or litter sizes would suggest that manifestation of cumulative reproductive costs is partly age‐dependent. For example, a study in female northern elephant seals showed reproductive success to decrease with previous reproductive frequency, but only in individuals aged between 11 and 15 years (Sydeman et al., 1991). These results are in line with both predictions of the general principle of allocation (Cody, 1966; Williams, 1966), as well as disposable soma theory of aging (Kirkwood, 1977; Kirkwood & Rose, 1991). However, we did not find evidence for age affecting cumulative effects, and therefore, our results neither support the hypothesis that cumulative costs are age‐dependent, nor the disposable soma theory in its classical sense. Although it might be worth taking a less age‐centered approach to disposable soma theory, that incorporates all allocation trade‐offs across females’ life spans, as opposed to only those among defined early and late ages (as suggested by Baudisch & Vaupel, 2012; also see Lemaître et al., 2015). If so, our result of decreasing reproduction probability in females with both high previous reproductive frequencies and average litter sizes could be considered as being in support of disposable soma theory. Indeed, from a less age‐centered perspective, the general absence of age‐related changes in female reproductive success also makes sense, if differences in current reproductive performance arise as a result of among‐individual differences in life‐history strategies (i.e., differences in reproductive frequencies and average litter sizes, generating differences in damage accumulation rates; McNamara & Houston, 1996; McNamara, Houston, Barta, Scheuerlein, & Fromhage, 2009), rather than as a result of chronological age per se (see Martin & Festa‐Bianchet, 2011; Kroeger et al., 2018).

Our study is in line with Hamel et al.’s prediction (2010), in that we found reproductive costs on current reproduction in a “long‐lived” species. However, negative effects of previous reproductive allocation were expressed as cumulative effects over the long‐term, rather than from one year to the next, which might be the rule rather than the exception in long‐lived species (Aubry et al., 2009). This may be particularly true for polytocous species, but further studies on polytocous mammals of varying life‐speeds are required to discern the generality of this statement. In addition, doing the same kinds of analyses in both sexes would be interesting to gain a more holistic understanding of cumulative costs within a given system. However, in yellow‐bellied marmots, reproductive systems between the sexes are fundamentally different: in males reproductive costs arise primarily through defending territories and females (Armitage, 2014, pp. 215–229), whereas in females, lactation is usually the costliest part of reproduction (Armitage, 2014, p. 226; Clutton‐Brock et al., 1989). These differences mean that a slightly different approach would be required to test cumulative reproductive costs in males, and we currently lack the required data to do so.

As we did not test for reproductive costs on survival, it is unclear whether reproductive costs on current reproduction were relatively easier to detect than reproductive costs on survival, and this requires further investigation. Finally, transgenerational studies would be interesting to elucidate whether cumulative reproductive costs are transferred to the offspring, reflected as decreases in offspring fitness components.

## CONFLICT OF INTEREST

None declared.

## AUTHORS’ CONTRIBUTIONS

S.B.K., J.G.A.M., and J.M.R. designed the study. S.B.K. analyzed the data and wrote the manuscript, with contributions from all authors.

## DATA ACCESSIBILITY

Data for this study have been archived in the Dryad Digital Repository https://doi.org/10.5061/dryad.9mk47dg.
