# Supplementary material for: Cumulative reproductive costs on current reproduction in a wild polytocous mammal
Source: Ecol Evol. 2018 Nov 14;8(23):11543–53. doi: 10.1002/ece3.4597 (PMC6303762; doi:10.1002/ece3.4597)
Supplement: Supplementary file 1 [file ECE3-8-11543-s001.docx]

**Appendix 1**

**Figure S1.** Correlations between the number of previous reproductive events and weaned offspring, previous reproductive frequency (PRF), previous average litter size (PALS) and female linear and quadratic age.

**Figure S2.** Distribution of (a) birth years and (b) observation years, of female yellow-bellied marmots in the data set. Earliest birth year = 1962, latest birth year = 2007. Earliest observation year = 1966, latest observation year = 2014.

**
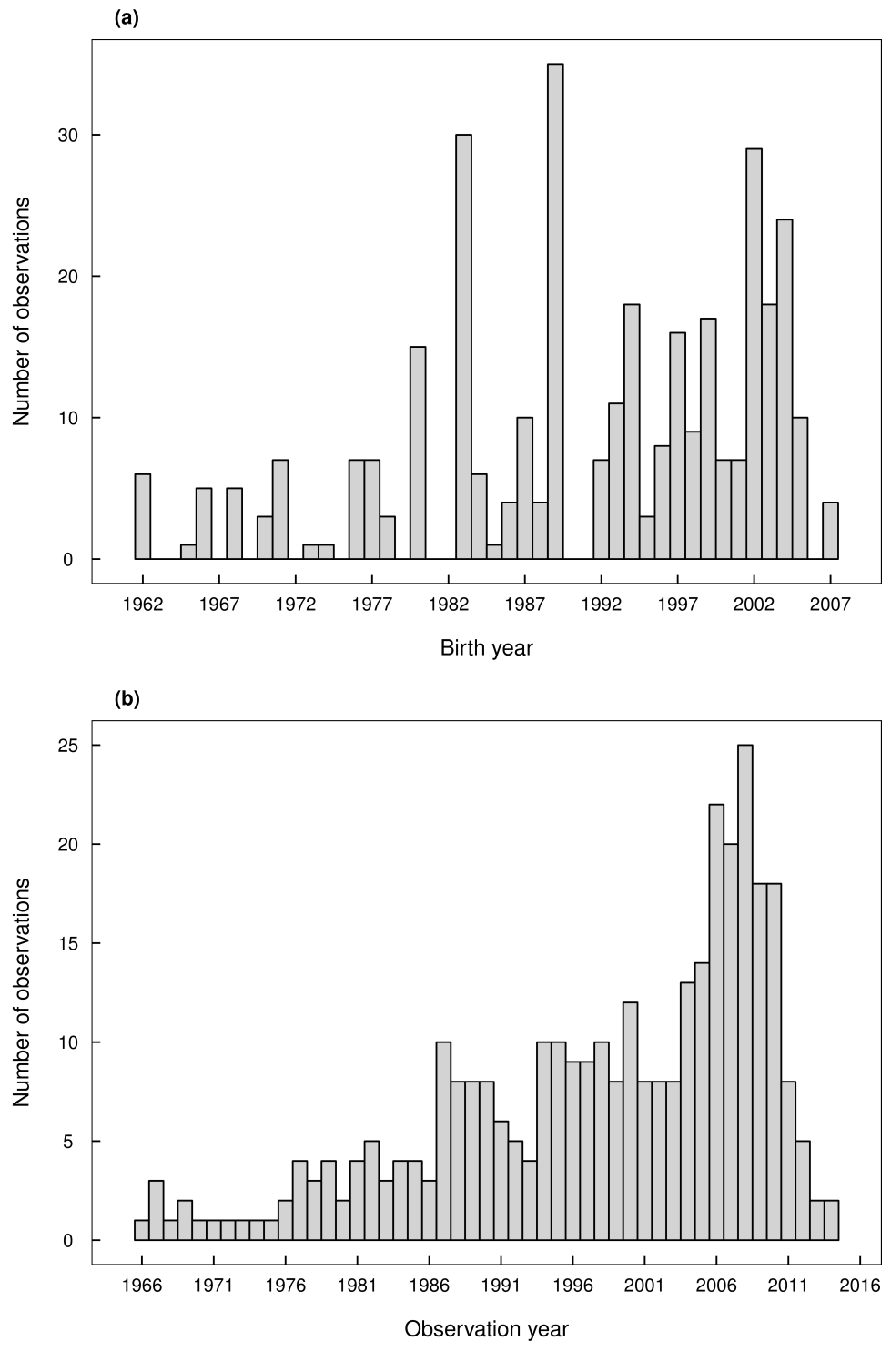
**

**Figure S3.** Distributions of (a) litter size, highlighting observations of defined reproducing (non-patterned) versus non-reproducing (diagonal pattern) female marmots; and (b-c) measures of previous cumulative reproduction: previous reproductive frequency (PRF), the proportion of years in which a female weaned pups since her first successful reproduction, excluding the current year; and previous average litter size (PALS), the total number of previously weaned pups divided by the total number of successful reproductive events up to and excluding the current year. Minimum litter size = 0 (non-reproducing observations; n = 156), maximum litter size = 9 (observations of successful reproduction: litter size > 0; n = 183). Minimum PRF value = 0.10, maximum PRF value = 1.00; minimum PALS value = 1, maximum PALS value = 8. Total sample size = 339 observations for 108 females.

**
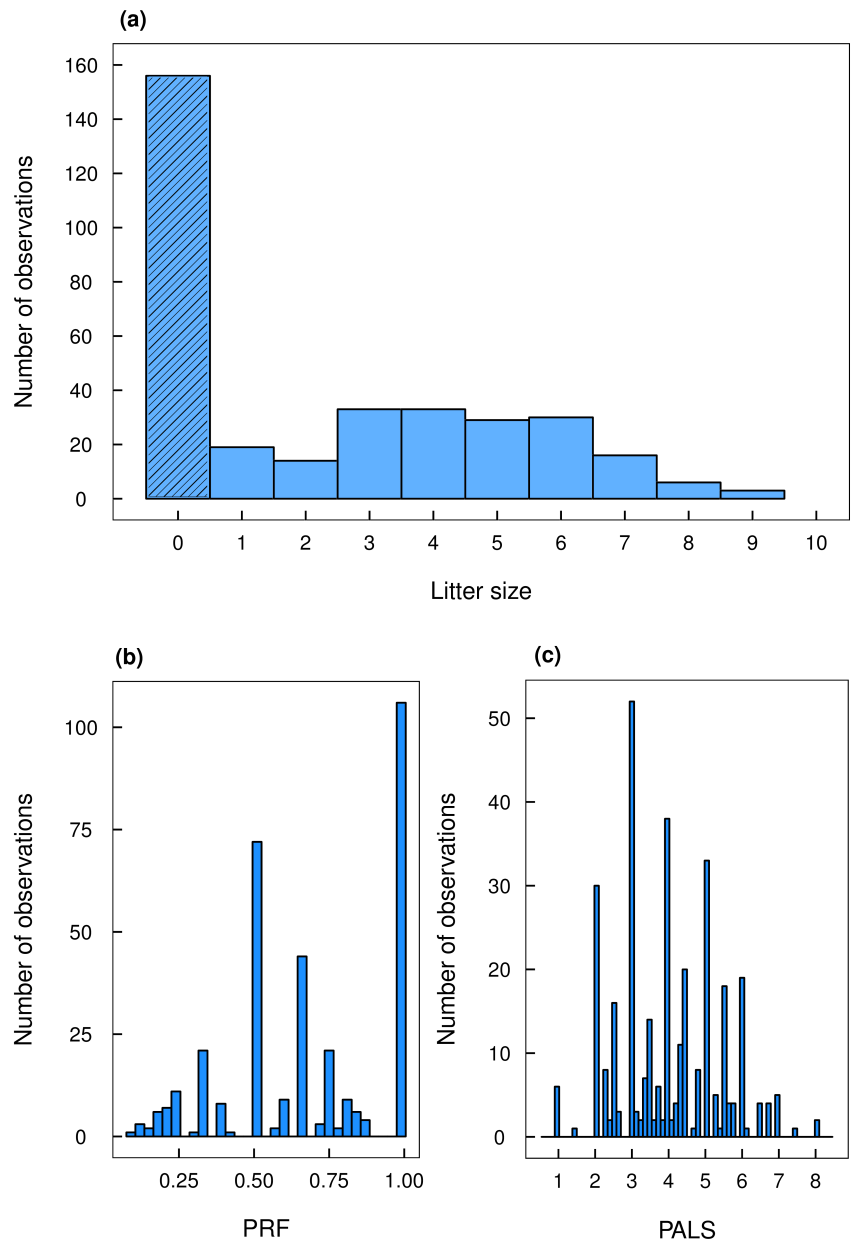
**

**Figure S4.** Distributions of numbers of observations (i.e. sample sizes) of (a) reproducing and (b) non-reproducing individuals at each age, and (c) litter sizes, of female yellow-bellied marmots in the data set, living down-valley (grey) and up-valley (white).

**
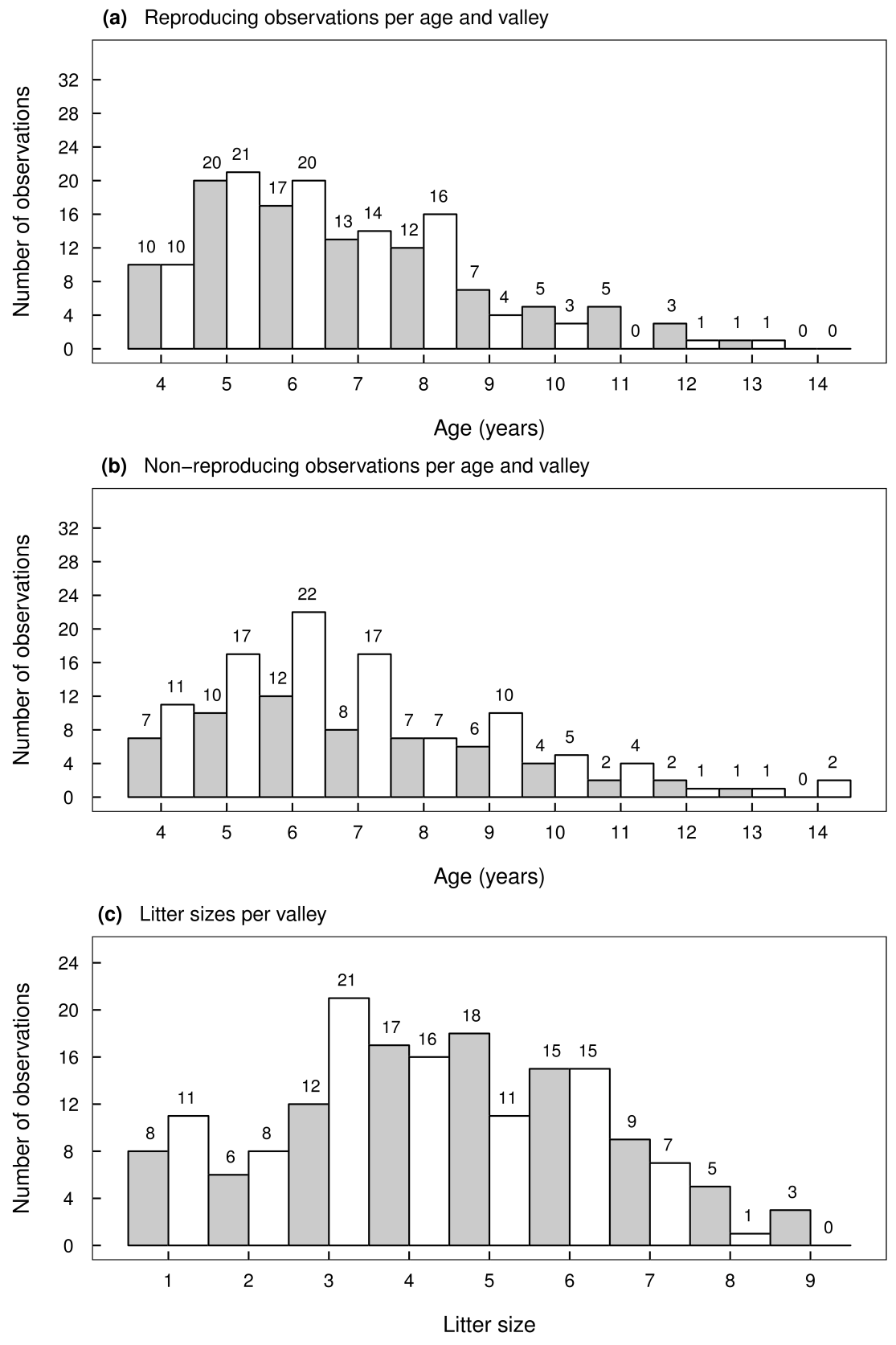
**

**Figure S5.** Relationships between (a) previous reproductive frequency (PRF) and years of reproductive activity and (b) previous average litter size (PALS) and number of successful reproductive events. Panel (a) shows the intrinsic structural relationship between PRF and years of reproductive activity that arises because PRF is defined as the total number of times a female has successfully weaned pups in the past, divided by the number of years since her first weaned litter (excluding the current year). For example, females that have been reproductively active for 3 years can only have 2 years of previous reproductive activity, thus they can only have PRF values of 0.5 or 1. However, despite the structural constraints, there is still considerable variation in PRF within each level of years of reproductive activity. Panel (b) shows that PALS varied substantially among females with different numbers of successful reproductive events (i.e. at least 1 weaned pup). PRF and PALS cannot be 0 as the data set is restricted to females that have had at least 1 successful reproductive event.


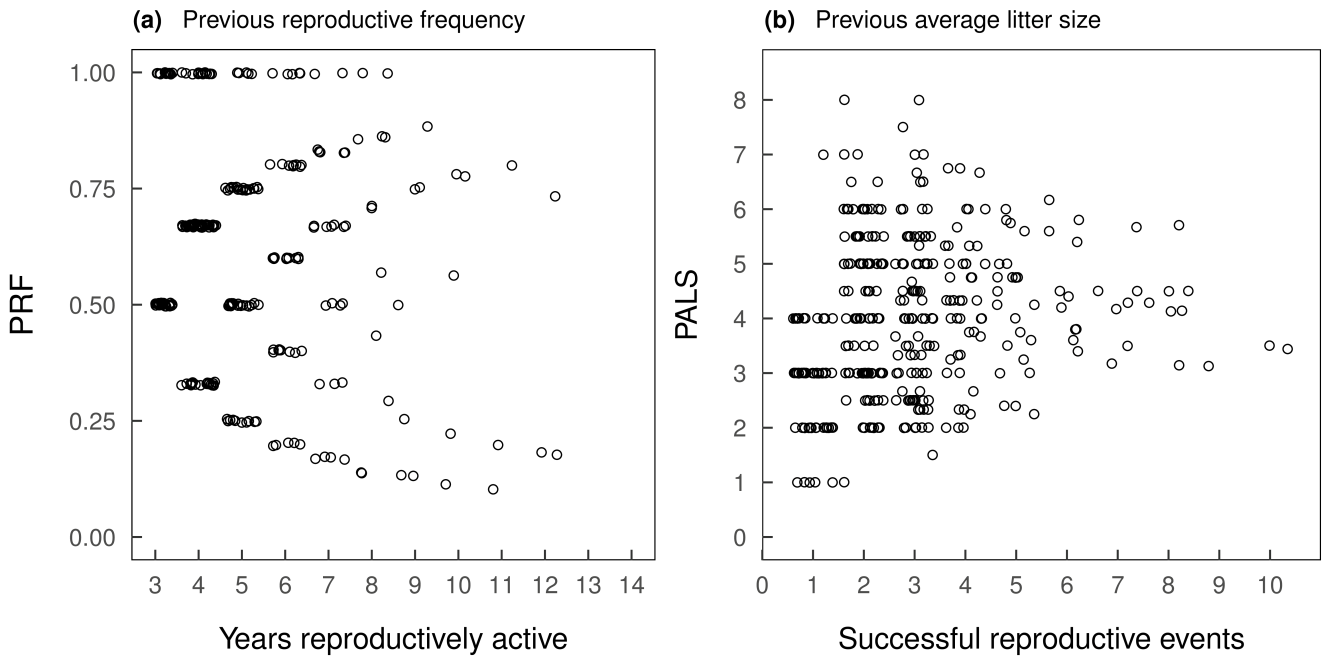


**Figure S6.** Relationships between (a) previous reproductive frequency (PRF) and reproduction last year (RLY), (b) previous average litter size (PALS) and RLY, and (c) PALS and PRF. Panel (a) shows that some females that reproduced the previous year and some females that did not reproduce the previous year had high or low values for PRF. However a PRF of 1.0 was only possible in females that reproduced the previous year, and they have to have successfully weaned pups in at least 2 previous reproductive attempts (data set includes only individuals from their third year of reproductive activity onwards). The pattern in (b) is similar but there were more possible values for PALS within each level of RLY than there were for PRF, because even if individuals had reproduced every year since sexual maturity, they may have only had small litters, resulting in low values for PALS. Panel (c) shows that there is considerable variation of PALS within each level of PRF, and the two variables only explained 5% of the variation in each other (R^2^ = 0.05).

**
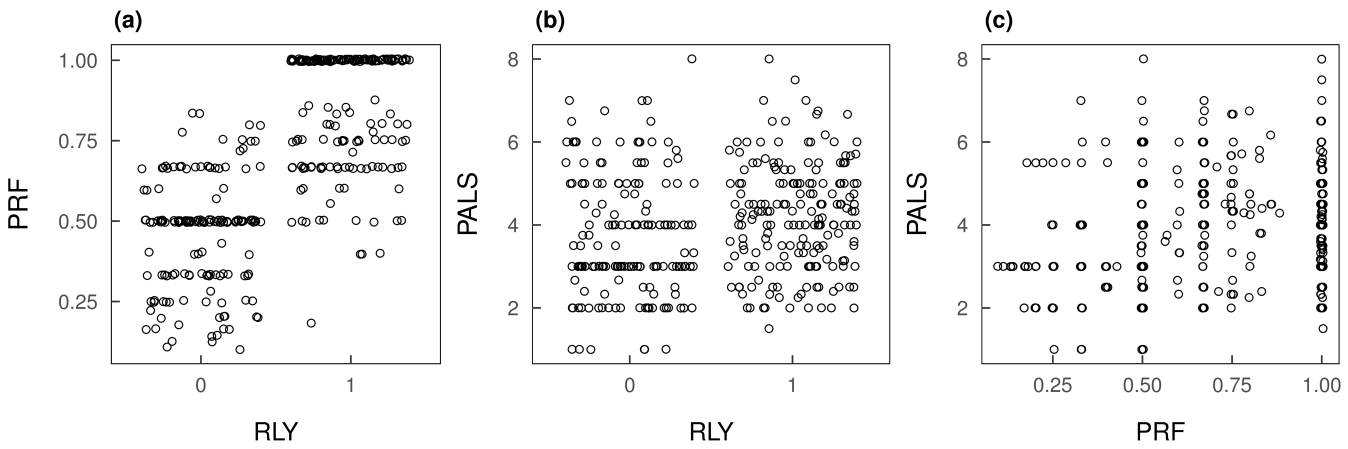
**

**Figure S7.** Current reproduction probability in relation to previous average litter size and previous reproductive frequency, represented as 2D images. Lines (and 95% confidence intervals) are the predictions using the (red) 75% and (blue) 25% quantiles of (a) previous reproductive frequency and (b) previous average litter size. Points show the raw data.

**
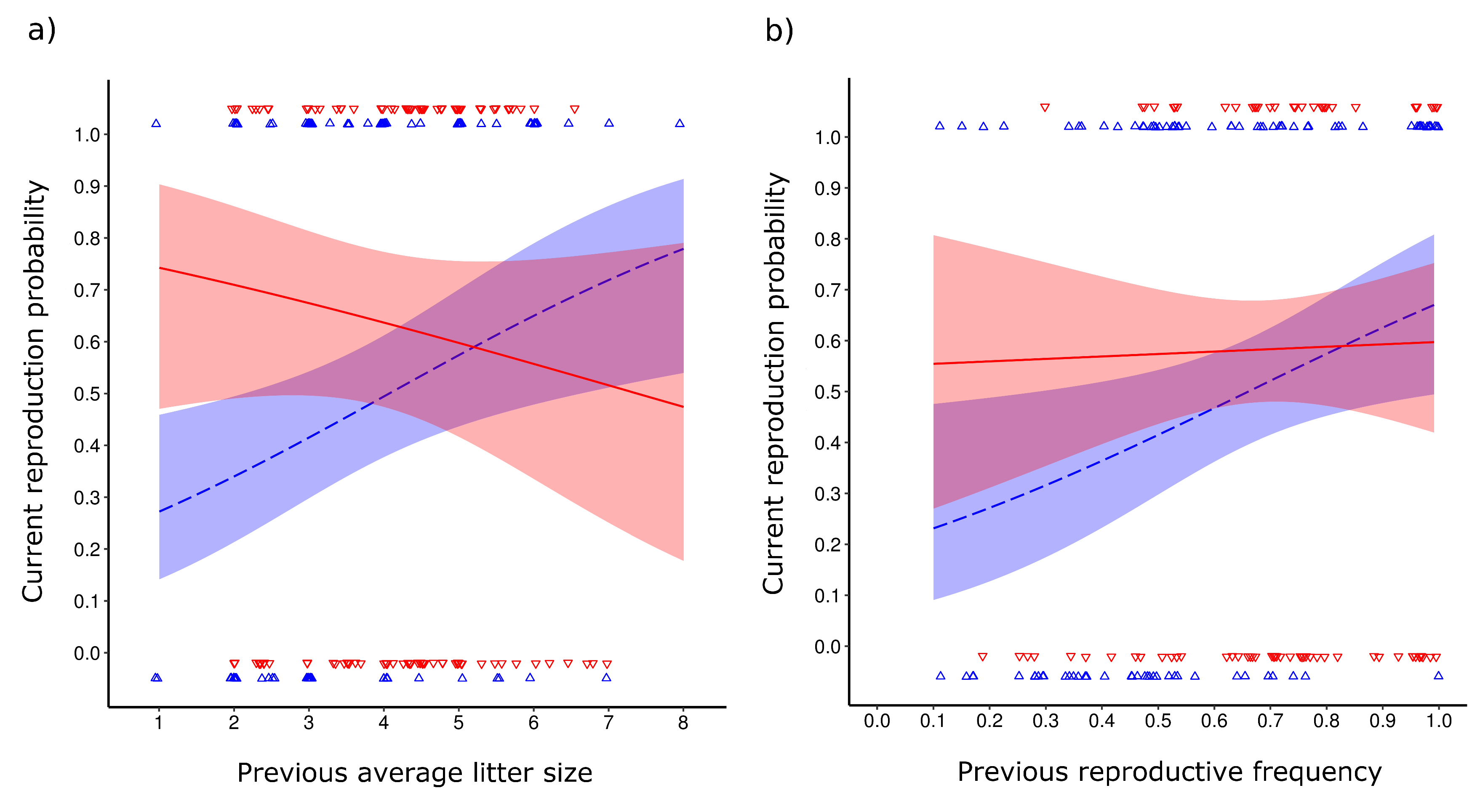
**

**Table S1.** Generalised linear mixed-effects model estimating effects of previous short-term reproduction (LYL, last year litter size) and cumulative long-term reproduction (PRF, previous reproductive frequency; and PALS, previous average litter size), age, valley, age at first reproduction (AFR) and number of sexually mature daughters living in the same colony (Mat_daughters) on current reproduction probability of female yellow-bellied marmots. This model’s structure is identical to the structure of the model reported in Table 1, except that it includes the short-term previous reproduction term LYL, instead of RLY, reproduced last year. The reference level for valley is [down]. Random effects variances are 0.00, 0.69 and 0.11 for ‘female identity’, ‘year observed’ and ‘cohort’ respectively. Estimated effect sizes are reported with standard errors (S.E.) and z-test statistics (z). Significant terms are shown in bold.

| Fixed effect | Estimate | S.E. | z | p-value |
| --- | --- | --- | --- | --- |
|  |  |  |  |  |
| Intercept | 0.37 | 0.28 | 1.31 | 0.190 |
| LYL | 0.00 | 0.08 | 0.04 | 0.967 |
| PRF | 1.01 | 0.84 | 1.20 | 0.228 |
| PALS | 0.15 | 0.12 | 1.26 | 0.209 |
| Age | -4.04 | 3.16 | -1.28 | 0.202 |
| Age² | -3.46 | 2.65 | -1.31 | 0.191 |
| Valley[up] | -0.28 | 0.32 | -0.87 | 0.383 |
| AFR | 0.08 | 0.17 | 0.49 | 0.622 |
| Mat_daughters | 0.17 | 0.13 | 1.35 | 0.177 |
| **PALS x PRF** | **-1.07** | **0.43** | **-2.49** | **0.013** |

**Table S2.** Generalised linear mixed-effects model estimating effects of previous short-term reproduction (LYL, last year litter size) and cumulative long-term reproduction (PRF, previous reproductive frequency; and PALS, previous average litter size), age, valley, age at first reproduction (AFR) and number of sexually mature daughters living in the same colony (Mat_daughters) on current litter size of female yellow-bellied marmots. This model’s structure is identical to the structure of the model reported in Table 2, except that it includes the short-term previous reproduction term LYL, instead of RLY, reproduced last year. The reference level for valley is [down]. Random effects variances are 0.00, 0.00 and 0.01 for ‘female identity’, ‘year observed’ and ‘cohort’ respectively. Estimated effect sizes are reported with standard errors (S.E.) and z-test statistics (z). Significant terms are shown in bold.

| Fixed effect | Estimate | S.E. | z | p-value |
| --- | --- | --- | --- | --- |
|  |  |  |  |  |
| **Intercept** | **1.49** | **0.06** | **24.10** | **<0.001** |
| LYL | 0.01 | 0.02 | 0.30 | 0.762 |
| PRF | 0.20 | 0.23 | 0.90 | 0.369 |
| PALS | 0.06 | 0.03 | 1.93 | 0.053 |
| Age | -0.32 | 0.70 | -0.46 | 0.646 |
| Age² | -0.20 | 0.55 | -0.37 | 0.709 |
| Valley[up] | -0.09 | 0.09 | -1.04 | 0.300 |
| AFR | -0.07 | 0.05 | -1.52 | 0.130 |
| Mat_daughters | 0.01 | 0.04 | 0.36 | 0.719 |

**Table S3.** Generalised linear mixed-effects model estimating effects of previous cumulative long-term reproduction (PRF, previous reproductive frequency; and PALS, previous average litter size), age, valley, age at first reproduction (AFR) and number of sexually mature daughters living in the same colony (Mat_daughters) on current reproduction probability of female yellow-bellied marmots. This model’s structure is identical to the structure of the model reported in Table 1, except that it excludes the short-term previous reproduction term RLY; reproduced last year. The reference level for valley is [down]. Random effects variances are 0.00, 0.69 and 0.11 for ‘female identity’, ‘year observed’ and ‘cohort’ respectively. Estimated effect sizes are reported with standard errors (S.E.) and z-test statistics (z). Significant terms are shown in bold.

| Fixed effect | Estimate | S.E. | z | p-value |
| --- | --- | --- | --- | --- |
|  |  |  |  |  |
| Intercept | 0.37 | 0.28 | 1.32 | 0.188 |
| PRF | 1.03 | 0.63 | 1.65 | 0.099 |
| PALS | 0.15 | 0.11 | 1.40 | 0.162 |
| Age | -4.01 | 3.05 | -1.31 | 0.189 |
| Age² | -3.47 | 2.63 | -1.32 | 0.186 |
| Valley[up] | -0.27 | 0.31 | -0.88 | 0.381 |
| AFR | 0.08 | 0.17 | 0.50 | 0.621 |
| Mat_daughters | 0.17 | 0.13 | 1.36 | 0.173 |
| **PALS x PRF** | **-1.06** | **0.41** | **-2.60** | **0.009** |

**Table S4.** Generalised linear mixed-effects model estimating effects of previous cumulative long-term reproduction (PRF, previous reproductive frequency; and PALS, previous average litter size), age, valley, age at first reproduction (AFR) and number of sexually mature daughters living in the same colony (Mat_daughters) on current litter size of female yellow-bellied marmots. This model’s structure is identical to the structure of the model reported in Table 2, except that it excludes the short-term previous reproduction term RLY; reproduced last year. The reference level for valley is [down]. Random effects variances are 0.00, 0.00 and 0.01 for ‘female identity’, ‘year observed’ and ‘cohort’ respectively. Estimated effect sizes are reported with standard errors (S.E.) and z-test statistics (z). Significant terms are shown in bold.

| Fixed effect | Estimate | S.E. | z | p-value |
| --- | --- | --- | --- | --- |
|  |  |  |  |  |
| **Intercept** | **1.49** | **0.06** | **24.03** | **<0.001** |
| PRF | 0.25 | 0.17 | 1.45 | 0.148 |
| **PALS** | **0.06** | **0.03** | **2.15** | **0.032** |
| Age | -0.26 | 0.66 | -0.39 | 0.699 |
| Age² | -0.23 | 0.54 | -0.42 | 0.675 |
| Valley[up] | -0.09 | 0.09 | -1.01 | 0.312 |
| AFR | -0.08 | 0.05 | -1.61 | 0.107 |
| Mat_daughters | 0.01 | 0.04 | 0.29 | 0.772 |

**Table S5.** Eliminated interaction terms of the generalised linear mixed-effects model presented in Table 1, estimating effects of previous short-term reproduction (RLY, reproduced last year) and cumulative long-term reproduction (PRF, previous reproductive frequency; and PALS, previous average litter size), age and valley on current reproduction probability of female yellow-bellied marmots. The reference levels for valley and RLY are [down] and [no] respectively. Estimated effect sizes are reported with standard errors (S.E.) and z-test statistics (z).

| Fixed effect | Estimate | S.E. | z | p-value |
| --- | --- | --- | --- | --- |
|  |  |  |  |  |
| Age x RLY[yes] | -0.18 | 5.34 | -0.03 | 0.973 |
| Age² x RLY[yes] | -8.28 | 4.94 | -1.68 | 0.094 |
| Age x PRF | 2.25 | 9.91 | 0.23 | 0.821 |
| Age² x PRF | -12.39 | 7.87 | -1.58 | 0.115 |
| Age x PALS | -1.58 | 2.23 | -0.71 | 0.480 |
| Age² x PALS | 4.55 | 2.36 | 1.93 | 0.053 |
| Valley[up] x RLY[yes] | -0.94 | 0.57 | -1.64 | 0.100 |
| Valley[up] x PRF | -1.97 | 1.20 | -1.63 | 0.103 |
| Valley[up] x PALS | 0.11 | 0.21 | 0.52 | 0.601 |

**Table S6.** Eliminated interaction terms of the generalised linear mixed-effects model presented in Table 2, estimating effects of previous short-term reproduction (RLY, reproduced last year) and cumulative long-term reproduction (PRF, previous reproductive frequency; and PALS, previous average litter size), age and valley on current litter size of female yellow-bellied marmots. The reference levels for valley and RLY are [down] and [no] respectively. Estimated effect sizes are reported with standard errors (S.E.) and z-test statistics (z).

| Fixed effect | Estimate | S.E. | z | p-value |
| --- | --- | --- | --- | --- |
|  |  |  |  |  |
| Age x RLY[yes] | 1.88 | 1.10 | 1.71 | 0.087 |
| Age² x RLY[yes] | -1.39 | 1.08 | -1.29 | 0.198 |
| Age x PRF | 3.41 | 2.07 | 1.65 | 0.100 |
| Age² x PRF | -1.11 | 1.64 | -0.68 | 0.497 |
| Age x PALS | -0.06 | 0.43 | -0.14 | 0.892 |
| Age² x PALS | -0.01 | 0.40 | -0.02 | 0.985 |
| Valley[up] x RLY[yes] | -0.18 | 0.16 | -1.12 | 0.263 |
| Valley[up] x PRF | -0.54 | 0.34 | -1.60 | 0.110 |
| Valley[up] x PALS | 0.00 | 0.06 | 0.00 | 0.997 |
| PRF x PALS | 0.01 | 0.12 | 0.08 | 0.938 |
